# Comorbidity management in inflammatory rheumatic diseases: a dual-perspective survey of patients and rheumatologists

**DOI:** 10.1186/s13075-026-03924-5

**Published:** 2026-10-23

**Authors:** Vanessa Bartsch, Basel Habboub, Johannes Knitza, Mathias Ausserwinkler, Stefan Knop, Georg Schett, Axel J. Hueber

**Affiliations:** 1 Division of Rheumatology, Klinikum Nürnberg, Paracelsus Medical University, Prof.-Ernst-Nathan-Str. 1, Nuremberg, 90419 Germany; 2 Department of Geriatrics, Klinikum Nürnberg, Paracelsus Medical University, Nuremberg, Germany; 3 Institute for Digital Medicine, Philipps University Marburg and University Hospital of Giessen and Marburg, Marburg, Germany; 4 First Department of Medicine, Uniklinikum Paracelsus Medical University Salzburg, Landeskrankenhaus, Austria; 5 Department of Internal Medicine 5, Klinikum Nürnberg, Paracelsus Medical University, Nuremberg, Germany; 6 Department of Internal Medicine 3 - Rheumatology and Immunology, Friedrich Alexander University Erlangen-Nuremberg and Universitätsklinikum Erlangen, Erlangen, Germany

**Keywords:** Inflammatory rheumatic diseases, Ccomorbidity management, EULAR points to consider, Vaccinations, Cardiovascular risk, Malignancies, Depression, Osteoporosis

## Abstract

**Background:**

Comorbidities are major contributors to morbidity and mortality in patients with inflammatory rheumatic diseases (IRDs). We assessed real-world implementation of European Alliance of Associations for Rheumatology (EULAR) points to consider for comorbidity management and perceptions of responsibility.

**Methods:**

We conducted a cross-sectional anonymous online survey among patients with IRDs and rheumatologists in Germany. Two questionnaires assessed demographics, perceived responsibility for comorbidity management, and implementation of preventive measures across five domains: cardiovascular risk, infections, malignancies, depression, and osteoporosis. Responses were analysed using descriptive statistics and group comparisons (chi-square, Fisher’s exact, Mann–Whitney U tests).

**Results:**

A total of 2,213 patients with IRDs and 140 rheumatologists participated. Patient-reported implementation of comorbidity management in routine care was low across all domains (mean 28.8%). Rheumatologists reported substantially higher implementation than patients across all domains, including blood pressure monitoring (83.6% vs 33.8%) and vaccination checks (77.9% vs 33.2%). Most rheumatologists (69.3%) reported that no structured comorbidity screening was in place. Reported barriers included lack of time (91.4%), unclear responsibilities (48.6%), staff shortages (40.0%), and lack of reimbursement (35.0%). Rheumatologists favoured shared responsibility with general practitioners (GPs) for documentation (61.4%) and screening (48.6%), whereas treatment was more often assigned to GPs alone (60.7%). Patients more frequently assigned primary responsibility to GPs, particularly for cardiovascular risk management (72.6%) and vaccinations (73.8%).

**Conclusions:**

Comorbidity management in IRDs is poorly implemented in routine care, and perceptions of responsibility differ between patients and rheumatologists. Clearer role definitions, stronger guidance from rheumatology organisations, and practical tools are needed to improve implementation.

**Supplementary Information:**

The online version contains supplementary material available at https://doi.org/10.1186/s13075-026-03924-5.

## Background

Patients with inflammatory rheumatic diseases (IRDs) are at an increased risk of comorbidities, such as cardiovascular diseases, infections, malignancies, depression and osteoporosis [1–4]. This increased risk is closely related to the chronic inflammatory state underlying IRDs as well as to immunomodulatory therapies used to control disease activity [5]. Comorbidities negatively affect disease outcomes, treatment success, and mortality, highlighting the importance of systematic screening and appropriate management [6–10]. The elevated comorbidity burden in IRDs is reflected in several disease-specific recommendations that go beyond preventive strategies for the general population. For example, European Alliance of Associations for Rheumatology (EULAR) recommendations for cardiovascular risk management in IRDs acknowledge the increased cardiovascular risk associated with chronic inflammation and state that rheumatologists should ensure regular cardiovascular risk assessment. This includes evaluation of traditional cardiovascular risk factors such as blood pressure, lipid levels, smoking status, and diabetes, while management of these risk factors is often coordinated with primary care physicians [11]. Similarly, European Society of Cardiology (ESC) cardiovascular prevention guidelines recommend that, in addition to optimal control of inflammation, cardiovascular risk in patients with inflammatory diseases should be managed using the same preventive strategies applied to other high-risk populations [12]. For infection prevention, EULAR vaccination recommendations for patients with IRDs emphasise that vaccination status should be reviewed regularly by the rheumatology team and that vaccination programmes should be coordinated between rheumatologists and primary care physicians [13]. These recommendations include vaccination strategies that differ from those for the general population, such as targeted vaccination against pneumococcal disease and influenza. For malignancy prevention, patients with IRDs generally follow national cancer screening programmes, as no specific rheumatology-based screening recommendations exist. In contrast, osteoporosis prevention is partly addressed in national guidelines explicitly recognising inflammatory rheumatic diseases and glucocorticoid exposure as risk factors requiring targeted preventive strategies [14]. Beyond these domain-specific recommendations, the EULAR points to consider for reporting, screening and preventing comorbidities in chronic inflammatory rheumatic diseases emphasise that the rheumatology team has a central role in detecting and documenting comorbidities and should ensure that appropriate screening and preventive measures are addressed [5]. At the same time, these recommendations highlight the importance of close collaboration with general practitioners and other specialists to ensure that comorbidities are appropriately managed. Despite this guidance, real-world data suggest that comorbidity management remains suboptimally implemented in routine rheumatology care [15–19]. One possible explanation is the shared nature of comorbidity care, with responsibilities distributed between rheumatologists and general practitioners and not always clearly defined in routine practice. However, implementation in routine clinical practice remains unclear. In addition, this has rarely been assessed from both patient and rheumatologist perspectives. Therefore, we aimed to assess the implementation of EULAR points to consider and the perceived allocation of responsibility for comorbidity management in a dual-perspective survey of patients with IRDs and rheumatologists.

## Methods

We carried out a cross-sectional, anonymous online survey in Germany among patients with IRDs and rheumatologists. The survey included two separate web-based questionnaires, one for patients and one for rheumatologists (Supplementary Tables 1 and 2). Patients were eligible if they reported a diagnosis of an IRD and had regular appointments with a rheumatologist. Rheumatologists were eligible if they treated patients with IRDs in an outpatient setting. Questions were grouped into four categories, demographic data, perceived responsibility for comorbidity management, implementation of comorbidity management in clinical practice and views and barriers. Comorbidity screening was assessed using specific measures. Participants were recruited between 18 August 2025 and 2 January 2026 using a mixed strategy involving patient organisations and social media, including organisational websites, newsletters and social-media channels, IRD-focused Instagram accounts and closed Facebook groups, as described previously [20]. Rheumatologists were recruited through email invitations distributed by the German Society for Rheumatology and Clinical Immunology (DGRh) and the Professional Association of German Rheumatologists (BDRh), and by direct invitation of attendees at the German Rheumatology Congress in September 2025, where participants could complete the survey on study iPads. No financial incentives were offered for recruitment or participation. The survey was designed and reported in accordance with CHERRIES [21], with the completed checklist provided in Supplementary Table 3. The questionnaires were developed for this study based on prior literature and pretested by two study-team members for content, usability and technical functionality. Surveys were administered using REDCap (version 13.3.2) and Google Forms, with items presented in a fixed order and no adaptive questioning. Participation was voluntary and informed consent was obtained electronically. Access was open (link-based), with no identifiers, IP addresses, cookies or registration, all items required a response, incomplete (not fully submitted) questionnaires were excluded (Supplementary Fig. 1). Data were stored in the Nuvolus Cloud with access restricted to the study team. We excluded likely duplicate submissions with identical responses within 1 min. Because recruitment occurred via open links, the number of individuals exposed to the survey invitation or starting the survey could not be determined, therefore, view, participation and completion rates could not be calculated. The Philipps-University Marburg Research Ethics Committee confirmed that no ethical approval was required (Nr. 25–224 ANZ) for this anonymous survey study. All data were collected using multiple-choice survey items and are therefore presented as proportions. Categorical variables were compared using the chi-square test or Fisher’s exact test, as appropriate. Ordinal variables were analysed using the Mann–Whitney U test. Given the descriptive and exploratory nature of this survey, no correction for multiple testing was applied. Missing data were minimal, therefore, no imputation or additional adjustments were performed. No weighting was applied to adjust for sample representativeness. All statistical analyses were conducted using R version 4.5.2. During the preparation of this work, the authors used ChatGPT for language editing. After using this tool, the authors reviewed and edited the content as needed and take full responsibility for the content of the published article.

## Results

### Participant characteristics

We analysed 2,213 patient and 140 rheumatologist questionnaires. Among patients the majority (92.1%) were female (Table 1). The most frequently reported inflammatory rheumatic diseases were rheumatoid arthritis (45.5%), connective tissue diseases (29.3%) and psoriatic arthritis (28.0%). Most patients were aged 50–69 years (60.3%), followed by 30–49 years (29.5%). Among 140 rheumatologists 55.7% were female. All worked in outpatient clinics and years of experience in rheumatology were broadly distributed (Table 2). Weekly patient volume was typically 20 to 100 patients (65.7%), with 22.1% seeing > 100 patients per week. Most rheumatologists reported spending ≤ 30 min on initial consultations (58.6%) and ≤ 15 min on follow-up visits (60.7%). University-based rheumatologists were less experienced (*p* < 0.001), saw fewer patients per week (*p* < 0.001), and reported longer consultation times for initial (*p* = 0.006) and follow-up visits (*p* < 0.001) than those in non-university settings (Supplementary Table 4).

**Table 1:** Demographics of patient participants

Patients with IRDs (*n* = 2213), n (%)	
Years of age	
18–29	63 (2.8)
30–49	653 (29.5)
50–69	1334 (60.3)
≥ 70	163 (7.4)
Gender	
Female	2039 (92.1)
Male	169 (7.6)
Other	2 (0.1)
Prefer not to say	3 (0.1)
Country of residence	
Germany	2084 (94.2)
Austria	107 (4.8)
Switzerland	22 (1.0)
Rheumatic disease diagnosis°	
Rheumatoid arthritis	1007 (45.5)
Psoriatic arthritis	619 (28.0)
Fibromyalgia*	281 (12.7)
Systemic sclerosis	243 (11.0)
Axial spondyloarthritis	204 (9.2)
Sjögren’s disease	172 (7.8)
Systemic lupus erythematosus	163 (7.4)
Polymyalgia rheumatica	49 (2.2)
Other connective tissue diseases	48 (2.2)
Vasculitis	44 (2.0)
Myositis	20 (0.9)
Autoinflammatory syndromes	8 (0.4)
Time since diagnosis (years)	
< 1	76 (3.4)
1–5	739 (33.4)
6–10	453 (20.5)
≥ 11	945 (42.7)
Interval of rheumatology appointments	
Several times per month	6 (0.3)
About once per month	76 (3.4)
About every 3 months	1292 (58.4)
About every 6 months	603 (27.3)
About once per year	153 (6.9)
Less than once per year	83 (3.8)

°Multiple diagnoses per participant were permitted; percentages refer to % of participants

^*^Fibromyalgia reported secondary to another inflammatory rheumatic disease; participants

reporting fibromyalgia only were excluded

IRD = Inflammatory rheumatic disease

**Table 2:** Demographics of rheumatologist participants

Rheumatologists (*n* = 140), n (%)	
Years of experience in rheumatology	
< 5	40 (28.6)
5–10	28 (20.0)
11–20	33 (23.6)
> 20	39 (27.9)
Gender	
Female	78 (55.7)
Male	62 (44.3)
Country of practice	
Germany	136 (97.1)
Austria	2 (1.4)
Other	2 (1.4)
Work setting	
University hospital outpatient clinic	71 (50.7)
Office-based rheumatology practice	67 (47.9)
Other	2 (1.4)
Patients seen per week	
< 20	17 (12.1)
20–50	54 (38.6)
51–100	38 (27.1)
> 100	31 (22.1)
New patient consultation duration (minutes)	
≤ 15	12 (8.6)
16–30	70 (50.0)
31–45	35 (25.0)
45–60	21 (15.0)
> 60	2 (1.4)
Follow-up consultation duration (minutes)	
≤ 15	85 (60.7)
16–30	50 (35.7)
> 30	5 (3.6)

### Rheumatologists’ reported approach to comorbidity screening

The majority of rheumatologists (69.3%) reported that their clinics had no structured approach to comorbidity screening (Supplementary Fig. 2). Approximately half (52.9%) documented comorbidities at regular intervals. In most cases, rheumatologists themselves were primarily responsible for comorbidity screening (88.6%), less often medical assistants. Most rheumatologists (74.3%) reported that they actively take responsibility for managing comorbidities in their patients.

### Reported implementation of comorbidity management in daily practice

**Fig. 1:**
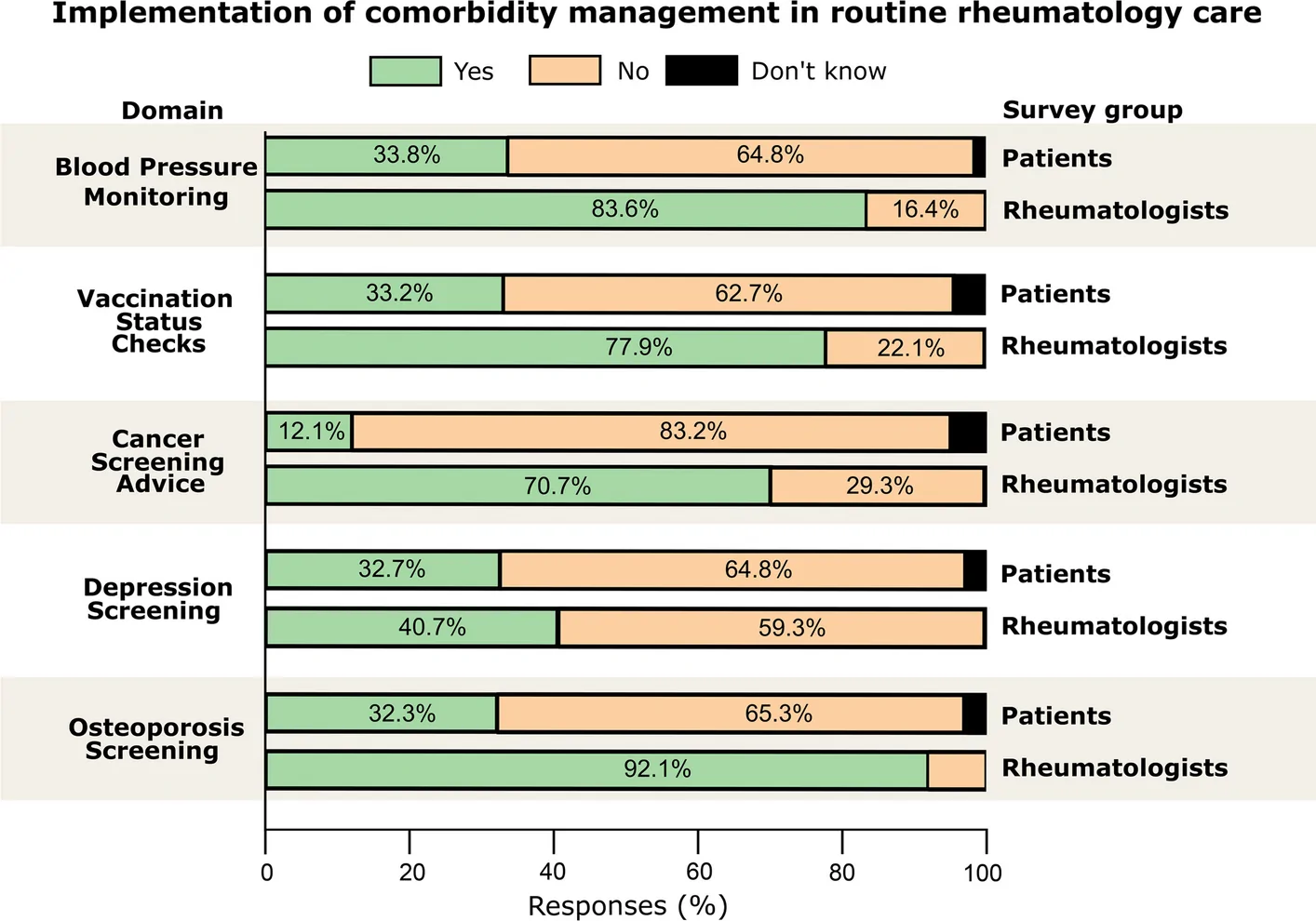
Reported implementation of comorbidity management measures in rheumatology care. Distribution of patient-reported experiences (*n* = 2213) and rheumatologist-reported practices (*n* = 140)

Mean patient-reported implementation of comorbidity management in clinical practice was 28.8% across all domains (Fig. 1). Across all analysed domains, rheumatologists reported higher implementation of comorbidity management measures than patients reported experiencing in routine care. The largest differences were observed in blood pressure monitoring (83.6% rheumatologists vs. 33.8% patients), checks of the vaccination status (77.9% vs. 33.2%), and osteoporosis screening (92.1% vs. 32.3%). Cancer screening checks and depression screening were implemented less often overall, with lower agreement between rheumatologists and patients, particularly for cancer screening advice (70.7% vs. 12.1%). The majority (59.1%) of patients disagreed with the statement that their rheumatologist has a good overview of their other health conditions in addition to their IRD. Moreover, different patient groups experienced different screening features (Supplementary Table 5). Whereas older patients were more likely to have their blood pressure measured and to be informed about preventive screening examinations (*p* < 0.001), patients with more frequent rheumatology visits were more likely to report being asked about depression and vaccination status and to report receiving information about preventive screening examinations. Patients with connective tissue diseases reported participation in preventive measures more frequently overall.

### Perceived responsibility for management of comorbidity domains

**Fig. 2:**
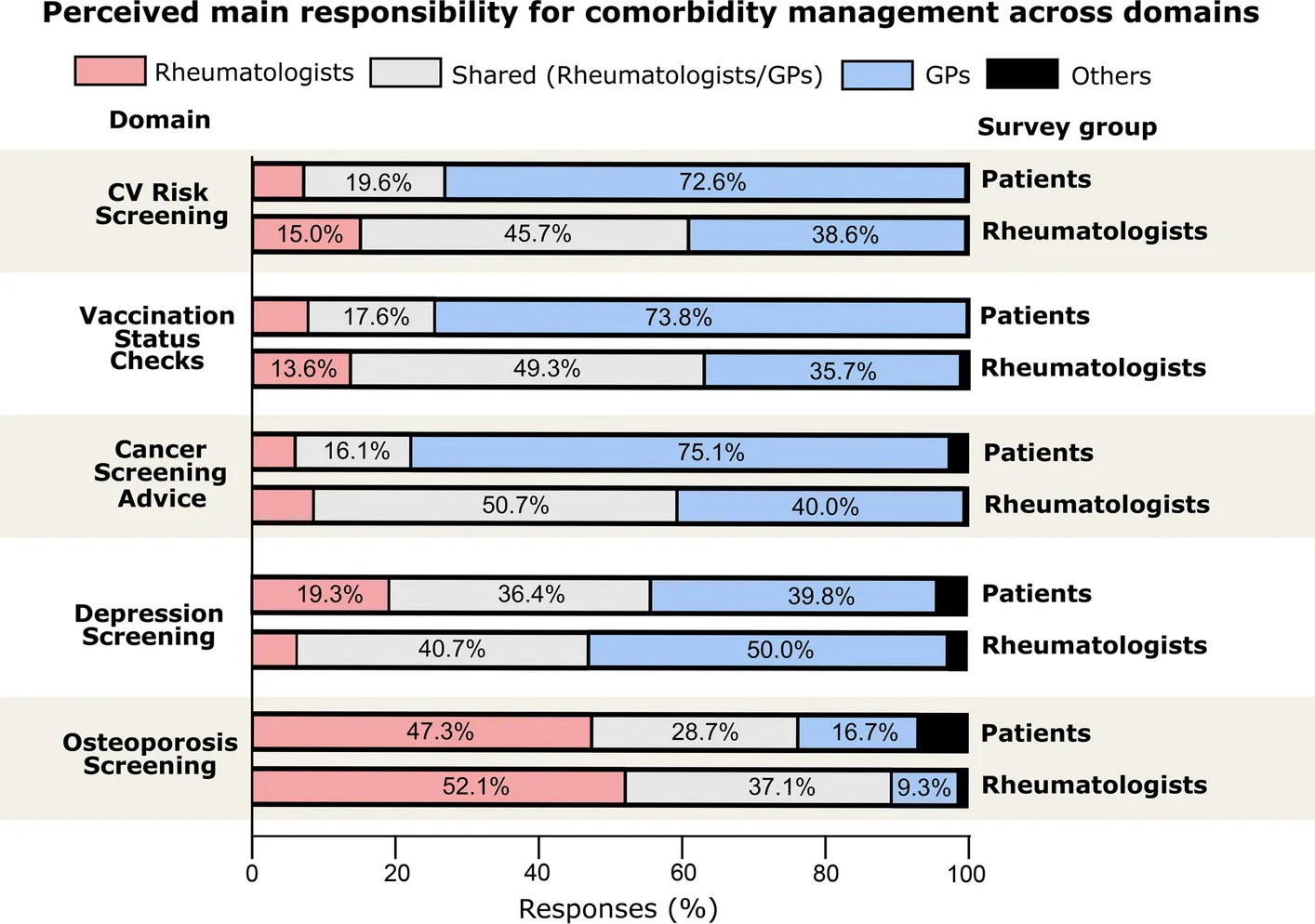
Perceived responsibility for comorbidity management in rheumatology care. Distribution of responses from patients (*n* = 2213) and rheumatologists (*n* = 140) regarding who should primarily manage key comorbidity domains. Bars show the proportion of respondents assigning responsibility to rheumatologists, general practitioners (GPs), or shared responsibility. CV = cardiovascular, CV Risk screening included blood pressure/lipid monitoring

Most rheumatologists reported that documentation and screening should be shared with general practitioners (61.4% and 48.6%, respectively), whereas responsibility for treatment was more often assigned to general practitioners (60.7%). When asked about responsibility for general health, most patients (88.7%) reported that responsibility should be shared between GPs and rheumatologists. The 116 patients who considered the rheumatologist to be primarily responsible attended consultations more frequently than those who perceived no role for the rheumatologist (*p* < 0.001, Supplementary Table 6). Perceived responsibility for the management of the five comorbidity domains differed between rheumatologists and patients (Fig. 2). Patients more frequently assigned primary responsibility to GPs, particularly for cardiovascular risk management (72.6%) and vaccinations (73.8%), while rheumatologists favored shared responsibility. For depression screening, more patients (19.3%) than rheumatologists (6.4%) considered this a rheumatologist’s responsibility. Osteoporosis screening was the only domain where patients (47.3%) and rheumatologists (52.1%) closely aligned in assigning primary responsibility to rheumatologists. A large proportion (89.1%) agreed that mental health should be a topic that can be discussed during the rheumatology consultations. Most patients (72.6%) agreed that they had sufficient time to ask questions during rheumatology consultations. Rheumatologists most frequently cited lack of time (91.4%), unclear responsibilities (48.6%), staff shortages (40.0%), and lack of incentives or reimbursement (35.0%) as barriers to screening implementation (Supplementary Fig. 3).

## Discussion

In this large cross-sectional survey, we compared patients’ and rheumatologists’ perspectives on comorbidity management in IRDs. Three main findings emerged. First, reported implementation of comorbidity management in clinical practice was low. Second, rheumatologists reported higher implementation of comorbidity management measures than patients reported experiencing in routine clinical practice. Third, patients more often assigned primary responsibility to GPs, whereas rheumatologists more frequently reported shared responsibility.

Overall, mean patient-reported implementation across comorbidity domains was 28.8%, indicating limited integration of comorbidity management into routine rheumatology care. This is in line with previous studies showing suboptimal implementation of preventive care in inflammatory rheumatic diseases [15, 18, 19].

In a Dutch survey of cardiovascular risk management in patients with rheumatoid arthritis, only 39% of patients reported being informed about their increased cardiovascular risk, despite rheumatologists reporting active involvement in preventive care [22]. Similarly, in a large RA registry, patients reported a 12-month prevalence of depressive symptoms of 11.7%, compared with only 1.0% reported by rheumatologists [23]. Part of this discrepancy may be explained by patient recall bias, differences in patient awareness of screening measures, or screening occurring outside rheumatology visits. However, since implementation of preventive care in patients with inflammatory rheumatic diseases has repeatedly been reported as suboptimal, these findings may also suggest that preventive measures are performed less consistently in routine practice than indicated by rheumatologist-reported implementation, potentially reflecting social desirability bias in physician reporting [15, 17, 18, 24–26]. The rheumatologist reported barriers to the implementation of comorbidity management in clinical practice are in line with other studies evaluating preventive care in rheumatology, including lack of time, unclear professional roles, and limited resources [22, 27]. Our results highlight a structural problem. Although many rheumatologists in our survey were willing to take at least some responsibility for documenting and screening comorbidities, this willingness did not appear to translate sufficiently into routine care. Current guidelines remain insufficiently specific regarding the allocation of responsibility for comorbidity management, and our survey showed that patients often rely on GPs. As a result, disease-specific risks, recommendations, and required screening or preventive measures may be overlooked. This is particularly likely when primary care physicians are not fully aware of the increased comorbidity burden in IRDs and the corresponding recommendations [28]. In addition, practical tools, such as checklists or app-based support, may further facilitate implementation in routine comorbidity management. In line with this, a recent study showed that systematic multimorbidity screening in patients with inflammatory rheumatic diseases increased the use of preventive medications and was associated with reduced hospitalisations [29]. Similarly, recording rates of cardiovascular risk factors increased substantially in a structured arthritis clinic model with clearly defined tasks for healthcare personnel [19]. Furthermore, structured nurse-led intervention or printed individualized checklists significantly improved screening and preventive management of comorbidities [15, 30].

### Strengths and limitations

This study has several strengths, including the large patient sample and the inclusion of both patient and rheumatologist perspectives on comorbidity management. However, some limitations should be considered. First, the survey was anonymous and based entirely on self-reported data, which did not allow external verification against medical records and may have introduced recall and reporting bias. In addition, 92% of patient respondents were female, which may limit representativeness, although this may partly reflect both disease epidemiology and participation patterns in survey-based IRD cohorts. High proportions of female participants (91%–94%) have also been reported in other German IRD surveys [24, 31]. Additionally, recruitment through patient organisations and online communities likely introduces participation bias toward highly engaged patients. Rheumatologists recruited through professional organisations and a national congress may likewise have had greater interest in or awareness of comorbidity management. As the two groups were recruited independently, differences between their responses should be interpreted as group-level rather than paired comparisons. Specific investigations, particularly bone-density measurement and individual cancer screening procedures, depend on individual risk or eligibility and may therefore not be indicated in every patient.

## Conclusion

In conclusion, our findings suggest that comorbidity management in inflammatory rheumatic diseases remains suboptimal, with marked discrepancies between patient-reported care and rheumatologist-reported practice and differing perceptions of responsibility. Clearer role definitions, stronger organisational guidance, and structured tools are needed to improve implementation. Further research should inform updated EULAR guidance and future recommendations.

## Supplementary Information

Supplementary Materials 1.

## Data Availability

The datasets generated and/or analysed during the current study are available from the corresponding author on reasonable request.

## Abbreviations

CTD: Connective tissue disease
CV: Cardiovascular
ESC: European Society of Cardiology
EULAR: European Alliance of Associations for Rheumatology
GP: General practitioner
IRD: Inflammatory rheumatic disease
