# Supplementary material for: Comorbidity management in inflammatory rheumatic diseases: a dual-perspective survey of patients and rheumatologists
Source: Arthritis Res Ther. 2026 Oct 23;28:187. doi: 10.1186/s13075-026-03924-5 (PMC13695290; doi:10.1186/s13075-026-03924-5)
Supplement: Supplementary file 1 [file 13075_2026_3924_MOESM1_ESM.docx]

Supplementary material

**Supplementary Figure 1.** Study Flow Chart

**Supplementary Figure 2.** Rheumatologists’ (n = 140) reported organisation of comorbidity screening in routine clinical care.

**Supplementary Figure 3.** Reported barriers to comorbidity management among rheumatologists. Responses from rheumatologists (n = 140) on barriers to managing comorbidities in patients with inflammatory rheumatic diseases. Multiple responses were permitted.

**Supplementary Table 1.** Patient questionnaire items (English translation)

| **Item** | **Section** | **English question (translated)** | **Response format / options** |
| --- | --- | --- | --- |
| 1 | Eligibility | Do you have an inflammatory rheumatic disease and do you regularly see a rheumatologist? | Single choice: Yes (continue with Question 2) / No (not eligible for study participation) |
| 2 | Demographics | In which country do you live? | Single choice: Germany / Austria / Switzerland / Other country |
| 3 | Demographics | How old are you? | Single choice: 18-29 years / 30-49 years / 50-69 years / 70 years or older |
| 4 | Demographics | What is your gender? | Single choice: Female / Male / Diverse / Prefer not to say |
| 5 | Demographics | How long have you had your rheumatic disease? | Single choice: Less than 1 year / 1-5 years / 6-10 years / More than 10 years |
| 6 | Demographics | What is your underlying rheumatic disease? | Multiple choice (select all that apply): Rheumatoid arthritis / Axial spondyloarthritis / Psoriatic arthritis / Systemic lupus erythematosus / Giant cell arteritis / Fibromyalgia / Polymyalgia rheumatica / Other (free text) |
| 7 | Demographics | How often do you see your rheumatologist? | Single choice: Several times per month / About once per month / About every 3 months / About every 6 months / About once per year / Less often |
| 8a | Perceived responsibility | Who should check blood pressure and blood lipids? | Matrix options: General practitioners / Rheumatologists / Both equally / Other |
| 8b | Perceived responsibility | Who should treat your blood pressure if needed? | Matrix options: General practitioners / Rheumatologists / Both equally / Other |
| 8c | Perceived responsibility | Who should treat your cholesterol if needed? | Matrix options: General practitioners / Rheumatologists / Both equally / Other |
| 8d | Perceived responsibility | Who should check your vaccination status? | Matrix options: General practitioners / Rheumatologists / Both equally / Other |
| 8e | Perceived responsibility | Who should vaccinate you (e.g., influenza)? | Matrix options: General practitioners / Rheumatologists / Both equally / Other |
| 8f | Perceived responsibility | Who should remind you about cancer screening examinations? | Matrix options: General practitioners / Rheumatologists / Both equally / Other |
| 8g | Perceived responsibility | Who should pay attention to your emotional/psychological strain? | Matrix options: General practitioners / Rheumatologists / Both equally / Other |
| 8h | Perceived responsibility | Who should take care of your bone health? | Matrix options: General practitioners / Rheumatologists / Both equally / Other |
| 8i | Perceived responsibility | Who should perform a bone density measurement? | Matrix options: General practitioners / Rheumatologists / Both equally / Other |
| 9 | Implementation in practice | How often is your blood pressure measured during your rheumatology appointment? | Single choice: At every visit / Occasionally / Never / Don't know |
| 10 | Implementation in practice | Who prescribes your blood pressure medication? | Single choice: Rheumatologist / General practitioner / Don't know / Not relevant (no known hypertension) |
| 11 | Implementation in practice | Will your blood cholesterol level be measured during your rheumatology appointment? | Single choice: Yes / Occasionally / No |
| 12 | Implementation in practice | Who prescribes lipid-lowering medication if your cholesterol is too high? | Single choice: Rheumatologist / General practitioner / Don't know / Not relevant (no known high cholesterol) |
| 13 | Implementation in practice | How often does your rheumatologist check your vaccination status (e.g., verbal check, vaccination booklet)? | Single choice: At every visit / Occasionally / Never / Don't know |
| 14 | Implementation in practice | Does your rheumatology practice offer vaccinations (e.g., influenza, shingles)? | Single choice: Yes / No / Don't know |
| 15 | Implementation in practice | How often are you asked in your rheumatology practice whether you attend relevant cancer screening examinations (e.g., breast cancer, skin cancer)? | Single choice: At every visit / Occasionally / Never / Don't know |
| 16 | Implementation in practice | Are you reminded in your rheumatology practice about cancer screening examinations relevant to you (e.g., breast cancer, skin cancer)? | Single choice: Yes / No / Don't know |
| 17 | Implementation in practice | How often has your rheumatologist asked whether you often feel sad or lacking motivation/drive? | Single choice: At every visit / Occasionally / Never / Don't know |
| 18 | Implementation in practice | Has your rheumatologist ever mentioned a bone density measurement? | Single choice: Yes / No / Don't know |
| 19 | Implementation in practice | Has a bone density measurement ever been performed in your rheumatology practice? | Single choice: Yes / No / Don't know |
| 20 | Attitudes and barriers | Do you agree with the following statement? "My rheumatologist also takes care of my general health (not only my rheumatic disease)." | 4-point Likert: Strongly agree / Somewhat agree / Somewhat disagree / Disagree |
| 21 | Attitudes and barriers | Do you agree with the following statement? "I have enough time to ask my rheumatologist questions during the consultation." | 4-point Likert: Strongly agree / Somewhat agree / Somewhat disagree / Disagree |
| 22 | Attitudes and barriers | Do you agree with the following statement? "I feel my rheumatologist has an overview of my other illnesses (e.g., cardiovascular, mental health)." | 4-point Likert: Strongly agree / Somewhat agree / Somewhat disagree / Disagree / Not relevant (no other illnesses) |
| 23 | Attitudes and barriers | Do you agree with the following statement? "I would like my mental health to be something that can be discussed during the rheumatology appointment." | 4-point Likert: Strongly agree / Somewhat agree / Somewhat disagree / Disagree |
| 24 | Attitudes and barriers | From your perspective, how much responsibility should the rheumatologist take for your general health? | Single choice: Primary responsibility / Shared responsibility with general practitioner / Only responsible for rheumatic disease / Don't know |

**Supplementary Table 2.** Rheumatologist questionnaire items (English translation)

| **Item** | **Section** | **English question (translated)** | **Response format / options** |
| --- | --- | --- | --- |
| 1 | Eligibility | Are you a physician and do you provide outpatient care to patients with inflammatory rheumatic diseases? | Single choice: Yes (continue) / No (study participation not possible) |
| 2 | Demographics | In which country do you primarily work? | Single choice: Germany / Austria / Switzerland / Other country |
| 3 | Demographics | What is your gender? | Single choice: Female / Male / Diverse / Prefer not to say |
| 4 | Demographics | In which setting do you primarily work? | Single choice: Office-based rheumatology practice / University hospital outpatient clinic/ Other |
| 5 | Demographics | How many years have you been working in rheumatology? | Single choice: <5 / 5-10 / 11-20 / >20 years |
| 6 | Demographics | Approximately how many rheumatology patients do you see per week? | Single choice: <20 / 20-50 / 51-100 / >100 |
| 7 | Demographics | What is your level of training? | Single choice: Rheumatology resident / Board-certified rheumatologist / Other |
| 8 | Demographics | On average, how much time do you have per patient (first visit/new patient)? | Single choice: <5 / 5-10 / 11-15 / 16-30 / 31-45 / 46-60 / >60 minutes |
| 9 | Demographics | On average, how much time do you have per patient (follow-up/returning patient)? | Single choice: <5 / 5-10 / 11-15 / 16-30 / 31-45 / 46-60 / >60 minutes |
| 10 | Perceived responsibility | Who should document comorbidities? | Matrix options: General practitioners / Rheumatologists / Both equally / Other |
| 10 | Perceived responsibility | Who should screen for comorbidities? | Matrix options: General practitioners / Rheumatologists / Both equally / Other |
| 10 | Perceived responsibility | Who should treat comorbidities? | Matrix options: General practitioners / Rheumatologists / Both equally / Other |
| 10 | Perceived responsibility | Who should screen for cardiovascular risk factors (e.g., blood pressure, cholesterol)? | Matrix options: General practitioners / Rheumatologists / Both equally / Other |
| 10 | Perceived responsibility | Who should treat cardiovascular risk factors? | Matrix options: General practitioners / Rheumatologists / Both equally / Other |
| 10 | Perceived responsibility | Who should remind patients about cancer screening examinations? | Matrix options: General practitioners / Rheumatologists / Both equally / Other |
| 10 | Perceived responsibility | Who should check vaccination status? | Matrix options: General practitioners / Rheumatologists / Both equally / Other |
| 10 | Perceived responsibility | Who should administer vaccinations? | Matrix options: General practitioners / Rheumatologists / Both equally / Other |
| 10 | Perceived responsibility | Who should screen for depression? | Matrix options: General practitioners / Rheumatologists / Both equally / Other |
| 10 | Perceived responsibility | Who should screen for osteoporosis? | Matrix options: General practitioners / Rheumatologists / Both equally / Other |
| 10 | Perceived responsibility | Who should treat osteoporosis? | Matrix options: General practitioners / Rheumatologists / Both equally / Other |
| 11 | Implementation in practice | Is there a structured approach to assessing comorbidities in your institution? | Single choice: Yes / No |
| 12 | Implementation in practice | How often are comorbidities systematically assessed in your institution? | Single choice: Only at initial visit / At regular intervals / At irregular intervals / Never |
| 13 | Implementation in practice | Who primarily performs comorbidity screening in your institution? | Single choice: Rheumatology medical assistants / Medical assistants / Physicians / Nurses / Nobody / Other |
| 14 | Implementation in practice | How often is blood pressure measured in your institution? | Single choice: At every visit / Occasionally / Never |
| 15 | Implementation in practice | Do you screen your patients for possible hypercholesterolemia? | Single choice: Yes / No / Sometimes |
| 16 | Implementation in practice | Do you prescribe antihypertensive medication yourself for your patients (e.g., ACE inhibitors)? | Single choice: Yes / No / Sometimes |
| 17 | Implementation in practice | Do you prescribe lipid-lowering medication yourself for your patients (e.g., statins)? | Single choice: Yes / No / Sometimes |
| 18 | Implementation in practice | Is your patients’ vaccination status recorded in your institution? | Single choice: Yes / No |
| 19 | Implementation in practice | Are vaccinations administered in your institution (e.g., influenza vaccination)? | Single choice: Yes / No |
| 20 | Implementation in practice | Is it recorded whether patients have participated in cancer screening examinations? | Single choice: Yes / No |
| 21 | Implementation in practice | Are all patients at your facility informed about relevant cancer screening examinations? | Single choice: Yes / No |
| 22 | Implementation in practice | How often do you use depression screening questionnaires (e.g., PHQ-2) in your institution? | Single choice: At every visit / At regular intervals / As needed / Never |
| 23 | Implementation in practice | Do you regularly check whether your patients need a bone density measurement? | Single choice: Yes / No |
| 24 | Implementation in practice | Is bone density measurement offered in your institution? | Single choice: Yes / No |
| 25 | Attitudes and barriers | Do you agree with the following statement: “Rheumatologists should actively take responsibility for managing comorbidities.” | 4-point Likert: Strongly agree / Somewhat agree / Somewhat disagree / Disagree |
| 26 | Attitudes and barriers | Do you agree with the following statement: “I actively take responsibility for managing comorbidities in my patients.” | 4-point Likert: Strongly agree / Somewhat agree / Somewhat disagree / Disagree |
| 27 | Attitudes and barriers | In your view, what are the biggest barriers to managing comorbidities? | Multiple choice (select all that apply): Lack of time / Unclear responsibilities / Lack of expertise / Low awareness / Lack of staff resources / Lack of digital tools / No reimbursement/incentives / Other |

**Supplementary Table 3.**  Checklist for Reporting Results of Internet E-Surveys (CHERRIES)

| **Category** | **CHERRIES item** | **Reporting** | **Location** |
| --- | --- | --- | --- |
| **Design** | Survey design | Cross-sectional anonymous online surveys. Separate patient and rheumatologist questionnaires. Open convenience sampling. | Methods |
| **IRB (Institutional Review Board) approval and informed consent process** | IRB approval | Ethics committee confirmed that formal approval was not required (25-224 ANZ). | Methods |
|  | Informed consent | Participation was voluntary. Survey completion constituted consent to scientific analysis and anonymised publication. | Methods |
|  | Data protection | Anonymous, no identifiers or IP addresses were collected, data was stored in Nuvolus Cloud with access restricted to the study team. | Methods |
| **Development and pretesting** | Development and testing | Questionnaires were developed for this study based on prior literature and pretested by two study-team members for content, usability, and technical functionality. | Methods |
| **Recruitment process and description of the sample having access to the questionnaire** | Open vs closed survey | Open, link-based surveys, no registration or authentication required. | Methods |
|  | Contact mode | Patients: Patient organisations and social media. Rheumatologists: Email invitations distributed by the German Society for Rheumatology and Clinical Immunology (DGRh) and the Professional Association of German Rheumatologists (BDRh) and direct recruitment of attendees at the German Rheumatology Congress 2025. | Methods |
|  | Advertising | Short invitation plus survey link, attendees of the German Rheumatology Congress 2025 were directly invited to complete the survey on study iPads. | Methods |
| **Survey administration** | Web / e-mail | Surveys administered using REDCap v13.3.2 and Google Forms. | Methods |
|  | Context | Patients were recruited through disease-related patient organisations and social media communities, rheumatologists through DGRh/BDRh email invitations and direct recruitment at the German Rheumatology Congress. | Methods |
|  | Mandatory / voluntary | Voluntary participation. | Methods |
|  | Incentives | None. | Methods |
|  | Time / date | 18 August 2025 to 2 January 2026. | Methods |
|  | Item randomisation | None, fixed item order. | Methods |
|  | Adaptive questioning | None, eligibility assessed at survey entry. | Methods |
|  | Number of items | 24 numbered patient items and 27 numbered rheumatologist items, items per page not recorded. | Suppl. Tables 1-2 |
|  | Number of screens / pages | Not recorded. | CHERRIES checklist |
|  | Completeness check | All items mandatory, unanswered required items prevented progression/submission. | Methods |
|  | Review step | No dedicated review/change step before final submission. | CHERRIES checklist |
| **Response rates** | Unique site visitor | Not recorded. | CHERRIES checklist |
|  | View rate | Not calculable, number of survey visitors/exposed users unavailable. | Methods |
|  | Participation rate | Not calculable, first-page visitor numbers unavailable. | Methods |
|  | Completion rate | Not calculable, number starting/consenting unavailable. | Methods |
| **Preventing multiple entries from the same individual** | Cookies | Not used for duplicate detection. | Methods |
|  | IP check | Not used, IP addresses were not collected. | Methods |
|  | Log-file analysis | Not performed, likely duplicates were identified as identical responses submitted within 1 minute. | Methods |
|  | Registration | None. | Methods |
| **Analysis** | Incomplete questionnaires | Incomplete questionnaires were excluded. | Methods |
|  | Atypical timestamps | Completion time alone was not an exclusion criterion, the 1-minute rule was used only for likely duplicates. | Methods |
|  | Statistical correction | No weighting or other adjustment for sample representativeness was performed. | Methods |

**Supplementary Table 4.** Characteristics of rheumatologists working in university hospital outpatient clinics compared with non-university settings.

| **Characteristic** | **Uni** N = 71*^1^* | **Non-Uni** N = 69*^1^* | **p-value** |
| --- | --- | --- | --- |
| Country of practice |  |  | 0.7 *^2^* |
| Other | 1 (1.4%) | 1 (1.4%) |  |
| Germany | 68 (96%) | 68 (99%) |  |
| Austria | 2 (2.8%) | 0 (0%) |  |
| Gender |  |  | 0.9 *^3^* |
| Male | 31 (44%) | 31 (45%) |  |
| Female | 40 (56%) | 38 (55%) |  |
| Years of experience in rheumatology |  |  | <0.001 *^4^* |
| <5 years | 31 (44%) | 9 (13%) |  |
| 5–10 years | 18 (25%) | 10 (14%) |  |
| 11–20 years | 12 (17%) | 21 (30%) |  |
| >20 years | 10 (14%) | 29 (42%) |  |
| Patients seen per week |  |  | <0.001 *^4^* |
| <20 | 12 (17%) | 5 (7.2%) |  |
| 20–50 | 38 (54%) | 16 (23%) |  |
| 51–100 | 14 (20%) | 24 (35%) |  |
| >100 | 7 (9.9%) | 24 (35%) |  |
| Training level |  |  | <0.001*^2^* |
| Rheumatology resident | 34 (48%) | 9 (13%) |  |
| Board-certified rheumatologist | 32 (45%) | 58 (84%) |  |
| Leadership / Other | 5 (7.0%) | 2 (2.9%) |  |
| New patient consultation duration |  |  | 0.006 *^4^* |
| <= 15 Minutes | 4 (5.6%) | 8 (12%) |  |
| 16 - 30 Minutes | 30 (42%) | 40 (58%) |  |
| 31 - 45 Minutes | 21 (30%) | 14 (20%) |  |
| >= 46 Minutes | 16 (23%) | 7 (10%) |  |
| Follow-up consultation duration |  |  | <0.001 *^4^* |
| <= 15 Minutes | 33 (46%) | 52 (75%) |  |
| 16 - 30 Minutes | 34 (48%) | 16 (23%) |  |
| > 30 Minutes | 4 (5.6%) | 1 (1.4%) |  |
| *^1^* n (%) | | | |
| *^2^* Fisher’s exact test; *^3^* Pearson’s Chi-squared test; *^4^* Wilcoxon rank sum test | | | |

**Supplementary Table 5.** Patients’ characteristics according to reported implementation of different comorbidity management domains

| **Blood pressure monitoring** |  |  |  |
| --- | --- | --- | --- |
| **Characteristic** | No N = 1,466*^1^* | Yes N = 747*^1^* | **p-value***^2^* |
| How often do you see your rheumatologist? |  |  |  |
| Several times per month | 2 (0.1%) | 4 (0.5%) | 0.82 *^2^* |
| About once per month | 41 (2.8%) | 35 (4.7%) |  |
| About every 3 months | 883 (60%) | 409 (55%) |  |
| About every 6 months | 375 (26%) | 228 (31%) |  |
| About once per year | 101 (6.9%) | 52 (7.0%) |  |
| Less often | 64 (4.4%) | 19 (2.5%) |  |
| Age |  |  | <0.001 *^2^* |
| 18 - 29 years | 41 (2.8%) | 22 (2.9%) |  |
| 30 - 49 years | 481 (33%) | 172 (23%) |  |
| 50 - 69 years | 864 (59%) | 470 (63%) |  |
| 70 years or older | 80 (5.5%) | 83 (11%) |  |
| Sex |  |  | <0.001 *^3^* |
| Male | 90 (6.2%) | 79 (11%) |  |
| Female | 1,372 (94%) | 667 (89%) |  |
| NA | 4 | 1 |  |
| RA |  |  | 0.003 *^3^* |
| Yes | 700 (48%) | 307 (41%) |  |
| No | 766 (52%) | 440 (59%) |  |
| SPA |  |  | <0.001 *^3^* |
| Yes | 558 (38%) | 226 (30%) |  |
| No | 908 (62%) | 521 (70%) |  |
| CTD |  |  | <0.001 *^3^* |
| Yes | 342 (23%) | 264 (35%) |  |
| No | 1,124 (77%) | 483 (65%) |  |
| *^1^*n (%), *^2^* Wilcoxon rank sum test; *^3^* Pearson's Chi-squared test | | | |

| **Cholesterol monitoring** |  |  |  |
| --- | --- | --- | --- |
| **Characteristic** | No N = 1,102*^1^* | Yes N = 1,111*^1^* | **p-value***^2^* |
| How often do you see your rheumatologist? |  |  | 0.16 *^2^* |
| Several times per month | 0 (0%) | 6 (0.5%) |  |
| About once per month | 32 (2.9%) | 44 (4.0%) |  |
| About every 3 months | 651 (59%) | 641 (58%) |  |
| About every 6 months | 277 (25%) | 326 (29%) |  |
| About once per year | 81 (7.4%) | 72 (6.5%) |  |
| Less often | 61 (5.5%) | 22 (2.0%) |  |
| Age |  |  | 0.65 *^2^* |
| 18 - 29 years | 29 (2.6%) | 34 (3.1%) |  |
| 30 - 49 years | 348 (32%) | 305 (27%) |  |
| 50 - 69 years | 650 (59%) | 684 (62%) |  |
| 70 years or older | 75 (6.8%) | 88 (7.9%) |  |
| Sex |  |  | <0.001*^3^* |
| Male | 60 (5.5%) | 109 (9.8%) |  |
| Female | 1,040 (95%) | 999 (90%) |  |
| NA | 2 | 3 |  |
| RA |  |  | 0.5 *^3^* |
| Yes | 509 (46%) | 498 (45%) |  |
| No | 593 (54%) | 613 (55%) |  |
| SPA |  |  | 0.067 *^3^* |
| Yes | 411 (37%) | 373 (34%) |  |
| No | 691 (63%) | 738 (66%) |  |
| CTD |  |  | <0.001 *^3^* |
| Yes | 265 (24%) | 341 (31%) |  |
| No | 837 (76%) | 770 (69%) |  |
| *^1^*n (%), *^2^* Wilcoxon rank sum test; *^3^* Pearson's Chi-squared test | | | |

| **Vaccination status checks** |  |  |  |
| --- | --- | --- | --- |
| **Characteristic** | No N = 1,479*^1^* | Yes N = 734*^1^* | **p-value***^2^* |
| How often do you see your rheumatologist? |  |  | < 0.001 *^2^* |
| Several times per month | 4 (0.3%) | 2 (0.3%) |  |
| About once per month | 46 (3.1%) | 30 (4.1%) |  |
| About every 3 months | 812 (55%) | 480 (65%) |  |
| About every 6 months | 421 (28%) | 182 (25%) |  |
| About once per year | 128 (8.7%) | 25 (3.4%) |  |
| Less often | 68 (4.6%) | 15 (2.0%) |  |
| Age |  |  | 0.79 *^2^* |
| 18 - 29 years | 36 (2.4%) | 27 (3.7%) |  |
| 30 - 49 years | 443 (30%) | 210 (29%) |  |
| 50 - 69 years | 889 (60%) | 445 (61%) |  |
| 70 years or older | 111 (7.5%) | 52 (7.1%) |  |
| Sex |  |  | 0.4 *^3^* |
| Male | 108 (7.3%) | 61 (8.3%) |  |
| Female | 1,368 (93%) | 671 (92%) |  |
| Unknown | 3 | 2 |  |
| RA |  |  | 0.9 *^3^* |
| Yes | 675 (46%) | 332 (45%) |  |
| No | 804 (54%) | 402 (55%) |  |
| SPA |  |  | 0.089 *^3^* |
| Yes | 542 (37%) | 242 (33%) |  |
| No | 937 (63%) | 492 (67%) |  |
| CTD |  |  | 0.043 *^3^* |
| Yes | 385 (26%) | 221 (30%) |  |
| No | 1,094 (74%) | 513 (70%) |  |
| *^1^*n (%), *^2^* Wilcoxon rank sum test; *^3^* Pearson's Chi-squared test | | | |
|  | | | |

| **Depression Screening** |  |  |  |
| --- | --- | --- | --- |
| **Characteristic** | No N = 1,489*^1^* | Yes N = 724*^1^* | **p-value** |
| How often do you see your rheumatologist? |  |  | 0.003 *^2^* |
| Several times per month | 3 (0.2%) | 3 (0.4%) |  |
| About once per month | 47 (3.2%) | 29 (4.0%) |  |
| About every 3 months | 853 (57%) | 439 (61%) |  |
| About every 6 months | 398 (27%) | 205 (28%) |  |
| About once per year | 113 (7.6%) | 40 (5.5%) |  |
| Less often | 75 (5.0%) | 8 (1.1%) |  |
| Age |  |  | 0.15 *^2^* |
| 18 - 29 years | 37 (2.5%) | 26 (3.6%) |  |
| 30 - 49 years | 432 (29%) | 221 (31%) |  |
| 50 - 69 years | 906 (61%) | 428 (59%) |  |
| 70 years or older | 114 (7.7%) | 49 (6.8%) |  |
| Sex |  |  | 0.031*^3^* |
| Male | 101 (6.8%) | 68 (9.4%) |  |
| Female | 1,384 (93%) | 655 (91%) |  |
| NA | 4 | 1 |  |
| RA |  |  | 0.2 *^3^* |
| Yes | 691 (46%) | 316 (44%) |  |
| No | 798 (54%) | 408 (56%) |  |
| SPA |  |  | 0.065 *^3^* |
| Yes | 547 (37%) | 237 (33%) |  |
| No | 942 (63%) | 487 (67%) |  |
| CTD |  |  | <0.001 *^3^* |
| Yes | 365 (25%) | 241 (33%) |  |
| No | 1,124 (75%) | 483 (67%) |  |
| *^1^*n (%), *^2^* Wilcoxon rank sum test; *^3^* Pearson's Chi-squared test | | | |

| **Cancer screening advice** |  |  |  |
| --- | --- | --- | --- |
| **Characteristic** | No N = 1,730*^1^* | Yes N = 483*^1^* | **p-value** |
| How often do you see your rheumatologist? |  |  | <0.001 *^2^* |
| Several times per month | 3 (0.2%) | 3 (0.6%) |  |
| About once per month | 54 (3.1%) | 22 (4.6%) |  |
| About every 3 months | 994 (57%) | 298 (62%) |  |
| About every 6 months | 470 (27%) | 133 (28%) |  |
| About once per year | 134 (7.7%) | 19 (3.9%) |  |
| Less often | 75 (4.3%) | 8 (1.7%) |  |
| Age |  |  | <0.001 *^2^* |
| 18 - 29 years | 51 (2.9%) | 12 (2.5%) |  |
| 30 - 49 years | 542 (31%) | 111 (23%) |  |
| 50 - 69 years | 1,020 (59%) | 314 (65%) |  |
| 70 years or older | 117 (6.8%) | 46 (9.5%) |  |
| Sex |  |  | <0.001 *^3^* |
| Male | 107 (6.2%) | 62 (13%) |  |
| Female | 1,620 (94%) | 419 (87%) |  |
| NA | 3 | 2 |  |
| RA |  |  | 0.008 *^3^* |
| Yes | 813 (47%) | 194 (40%) |  |
| No | 917 (53%) | 289 (60%) |  |
| SPA |  |  | 0.009 *^3^* |
| Yes | 637 (37%) | 147 (30%) |  |
| No | 1,093 (63%) | 336 (70%) |  |
| CTD |  |  | <0.001 *^3^* |
| Yes | 415 (24%) | 191 (40%) |  |
| No | 1,315 (76%) | 292 (60%) |  |
| *^1^*n (%) | | | |
| *^2^* Wilcoxon rank sum test; *^3^* Pearson's Chi-squared test | | | |

**Supplementary Table 6.** Patient characteristics according to perceived main responsibility of the rheumatologists for their general health

| **Characteristic** | **Primary responsibility** N = 116*^1^* | **No responsibility** N = 119*^1^* | **p-value** |
| --- | --- | --- | --- |
| Country |  |  | 0.13*^2^* |
| Germany | 103 (89%) | 112 (94%) |  |
| Austria | 11 (9.5%) | 4 (3.4%) |  |
| Switzerland | 2 (1.7%) | 3 (2.5%) |  |
| Age |  |  | 0.86*^3^* |
| 18 - 29 years | 4 (3.4%) | 2 (1.7%) |  |
| 30 - 49 years | 31 (27%) | 36 (30%) |  |
| 50 - 69 years | 74 (64%) | 74 (62%) |  |
| 70 years or older | 7 (6.0%) | 7 (5.9%) |  |
| Sex |  |  | 0.56*^4^* |
| Male | 8 (6.9%) | 6 (5.0%) |  |
| Female | 108 (93%) | 113 (95%) |  |
| Disease duration |  |  | 0.47*^3^* |
| Less than 1 year | 6 (5.2%) | 4 (3.4%) |  |
| 1 - 5 years | 39 (34%) | 39 (33%) |  |
| 6 - 10 years | 24 (21%) | 22 (18%) |  |
| More than 10 years | 47 (41%) | 54 (45%) |  |
| How often do you see your rheumatologist? |  |  | <0.001*^3^* |
| Several times per month | 2 (1.7%) | 0 (0%) |  |
| About once per month | 9 (7.8%) | 4 (3.4%) |  |
| About every 3 months | 74 (64%) | 55 (46%) |  |
| About every 6 months | 23 (20%) | 35 (29%) |  |
| About once per year | 6 (5.2%) | 12 (10%) |  |
| Less often | 2 (1.7%) | 13 (11%) |  |
| Rheumatologist takes care of my general health |  |  | <0.001*^3^* |
| Strongly disagree | 25 (22%) | 46 (39%) |  |
| Somewhat disagree | 38 (33%) | 46 (39%) |  |
| Somewhat agree | 30 (26%) | 22 (18%) |  |
| Strongly agree | 23 (20%) | 5 (4.2%) |  |
| Time to ask questions during the consultation |  |  | 0.53*^3^* |
| Strongly disagree | 7 (6.0%) | 8 (6.7%) |  |
| Somewhat disagree | 27 (23%) | 28 (24%) |  |
| Somewhat agree | 31 (27%) | 37 (31%) |  |
| Strongly agree | 51 (44%) | 46 (39%) |  |
| Rheumatologist has an overview of my other diseases |  |  | <0.001*^3^* |
| NA | 1 (0.9%) | 14 (12%) |  |
| Somewhat disagree | 32 (28%) | 41 (34%) |  |
| Somewhat agree | 27 (23%) | 29 (24%) |  |
| Strongly disagree | 28 (24%) | 30 (25%) |  |
| Strongly agree | 28 (24%) | 5 (4.2%) |  |
| Wish to discuss mental health with rheumatologist |  |  | <0.001*^3^* |
| Strongly disagree | 0 (0%) | 20 (17%) |  |
| Somewhat disagree | 9 (7.8%) | 26 (22%) |  |
| Somewhat agree | 27 (23%) | 48 (40%) |  |
| Strongly agree | 80 (69%) | 25 (21%) |  |
| RA |  |  | 0.9 *^4^* |
| Yes | 51 (44%) | 51 (43%) |  |
| No | 65 (56%) | 68 (57%) |  |
| SPA |  |  | 0.041 *^4^* |
| Yes | 33 (28%) | 49 (41%) |  |
| No | 83 (72%) | 70 (59%) |  |
| CTD |  |  | <0.001*^4^* |
| Yes | 43 (37%) | 21 (18%) |  |
| No | 73 (63%) | 98 (82%) |  |
| *^1^*n (%) | | | |
| *^2^*Fisher's exact test; *^3^* Wilcoxon rank sum test; *^4^* Pearson's Chi-squared test; | | | |
